# Inhibition of miR-200b Promotes Angiogenesis in Endothelial Cells by Activating The Notch Pathway

**DOI:** 10.22074/cellj.2021.7080

**Published:** 2021-03-01

**Authors:** Tie-Ying Qiu, Jin Huang, Li-Ping Wang, Bi-Song Zhu

**Affiliations:** 1 Clinical Nursing Teaching and Research Section of the Second Xiangya Hospital, Changsha 410011, P.R. China; 2 Organ Transplant Center, Xiangya Hospital, Central South University, Changsha 410008, P.R. China

**Keywords:** Angiogenesis, HUVEC Dysfunction, miR-200b, Notch Pathway

## Abstract

**Objective:**

Patients with diabetes mellitus frequently have chronic wounds or diabetic ulcers as a result of impaired wound healing, which may lead to limb amputation. Human umbilical vein endothelial cell (HUVEC) dysfunction also delays wound healing. Here, we investigated the mechanism of miR-200b in HUVECs under high glucose conditions and the potential of miR-200b as a therapeutic target.

**Materials and Methods:**

In this experimental study, HUVECs were cultured with 5 or 30 mM glucose for 48 hours. Cell proliferation was evaluated by CCK-8 assays. Cell mobility was tested by wound healing and Transwell assays. Angiogenesis was analyzed *in vitro* Matrigel tube formation assays. Luciferase reporter assays were used to test the binding of miR-200b with Notch1.

**Results:**

miR-200b expression was induced by high glucose treatment of HUVECs (P<0.01), and it significantly repressed cell proliferation, migration, and tube formation (P<0.05). Notch1 was directly targeted and repressed by miR-200b at both the mRNA and protein levels. Inhibition of miR-200b restored Notch1 expression (P<0.05) and reactivated the Notch pathway. The effects of miR-200b inhibition in HUVECs could be reversed by treatment with a Notch pathway inhibitor (P<0.05), indicating that the miR-200b/Notch axis modulates the proliferation, migration, and tube formation ability of HUVECs.

**Conclusion:**

Inhibition of miR-200b activated the angiogenic ability of endothelial cells and promoted wound healing through reactivation of the Notch pathway *in vitro*. miR-200b could be a promising therapeutic target for treating HUVEC dysfunction.

## Introduction

Diabetes mellitus (DM) and complications from having DM are a threat to global health. Over 400 million adults have DM worldwide. This number is estimated to reach 640 million by 2040 (1). Type 2 diabetes mellitus (T2DM), which accounts for over 90% of DM cases, and complications from having T2DM have contributed tremendously to the global mortality and disability of this disease (2, 3). Traditionally, the complications of DM have been divided into two groups: macrovascular complications (such as cardiovascular diseases) and microvascular complications (those affecting the retina or the nervous system). Complications are very common in T2DM patients; almost 50% of patients have microvascular complications, and 30% have macrovascular complications, with rates that vary in different countries (4). Diabetic skin ulcers are nonhealing and chronic wounds, and they are one of the most severe complications of D M(5), with up to 25% of DM patients developing these ulcers in their lifetime and 20% of these patients requiring amputations (6). Tremendous efforts have been made to explore the treatment for diabetic ulcers, including bioengineered skin substitutes and negative pressure dressings (7). However, there is still no effective therapeutic method.

Wound healing is a dynamic and complex process involving multiple cellular activities, including inflammation, proliferation, angiogenesis, and tissue remodeling (8). In diabetes, however, the healing process is impaired by an excessive inflammatory response and decreased angiogenesis (9). Studies have shown that enhancers of angiogenesis, such as growth factors, can facilitate the proliferation and migration of endothelial cells, accelerating the wound healing process in DM (10). The importance of the Notch signaling pathway in wound healing has been thoroughly demonstrated. There are four Notch receptors in mammals (Notch1, Notch2, Notch3, and Notch4), all of which are single-pass transmembrane receptor proteins. Moreover, mammals possess five ligands for the Notch pathway (Delta-like 1, 3, 5, Jagged 1, 2). Once the ligand binds to the extracellular domain of Notch proteins, they undergo proteolytic cleavage, leading to the release of the Notch intracellular domain (NICD), which enters the nucleus and acts as a transcription factor or forms a complex with other proteins to regulate the transcription of target genes (11, 12). The Notch signaling pathway plays a significant role in cell communication, regulating various biological processes during development and disease pathology (13, 14). Recent studies have found that Notch signaling promotes diabetic wound healing by regulating macrophage-mediated inflammation during the healing process (15). Additionally, the angiogenic ability of endothelial cells has been shown to be affected by Notch signaling, which could be due to the influence of vascular endothelial growth factor (VEGF) (16). The underlying molecular mechanism of Notch pathway-mediated wound healing is still unclear.

MicroRNAs (miRNAs) are small non-coding RNAs that are ~22 nt in length. miRNAs are post-transcriptional regulators that function by binding to the 3’ untranslated region (3’UTR) of target mRNAs and inducing translational repression or mRNA degradation (17). miRNAs play significant roles in diverse biological processes, and they are dysregulated in numerous diseases (18, 19). Several miRNAs have been discovered to regulate angiogenesis in tumors or during wound healing (20, 21). miR-200b belongs to the miR-200 family and is widely expressed in various cell types, including cancer cells, stem cells, and endothelial cells (22- 24). miR-200b has been demonstrated to regulate multiple cellular functions, such as migration, proliferation, and apoptosis (22). Moreover, inhibition of miR-200b has been linked with the promotion of angiogenesis by endothelial cells (25). However, the mechanism by which miR-200b acts and its downstream targets involved in the diabetic wound healing process are not quite clear

In this work, we tried to demonstrate that miR-200b could target the Notch pathway, leading to the suppression of angiogenesis *in vitro*. We also aimed to evaluate the therapeutic properties of miR-200b inhibitors in facilitating the diabetic wound healing process.

## Materials and Methods

### Cell culture and treatment

In this experimental study, HUVECs were obtained from ScienCell Research Laboratories (Carlsbad, CA, USA). The HUVECs were cultured in F-12K medium supplemented with 1% antibiotics (100 U/ml penicillin and 100 mg/ml streptomycin, Cat. 15240062, Life Technologies, USA) and were maintained at 37˚C in 5% CO_2_ . To mimic diabetic conditions, the HUVECs were incubated under high glucose (30 mM glucose, HG) conditions for 12, 24, and 48 hours. Cells treated with 5 mM glucose as normal glucose (NG) were used as controls. Cells were then harvested for subsequent assays. The research purposes under protocols were approved by Xiangya Hospital.

### Cell transfection

miRNA-negative controls and miR-200b inhibitors were purchased from GenePharma (Suzhou, China) and transfected into HUVECs at a concentration of 100 nM using Lipofectamine 2000 transfection reagent (Cat. 11668019, Invitrogen, USA) according to the manufacturer’s instructions. After 48 hours, the cells were used for subsequent experiments.

### Total RNA extraction and quantitative real-time PCR

Cells were dissolved in TRIzol reagent (Cat. 15596018, Invitrogen, USA), and total RNA was obtained according to the manufacturer’s protocol. The RNA was then tested for quality and synthesized into cDNA using an iScript cDNA Synthesis Kit (Cat. 1708891, Bio-Rad, USA). qRTPCR was performed using SYBR Green Supermixes (Cat. 1708882, Bio-Rad, USA). GAPDH and U6 were used as endogenous controls for normalization. Relative levels of expression were normalized and analyzed using the 2^−ΔΔCt^ method. Primer sequences are listed in Table 1.

### Western blot analysis

Cells were washed with cold PBS and incubated with lysis buffer on ice for 30 minutes. Then, the cells were scraped, and after centrifugation, the supernatant containing the lysate was collected and stored at -80˚C. A BCA assay kit (Cat. 5000001, Bio-Rad, USA) was used to determine protein concentrations. Protein samples were denatured and then separated by SDS-PAGE and transferred to PVDF membranes (Cat. IPVH00010, Millipore, USA). After blocking with nonfat milk for 1 hour, membranes were incubated overnight at 4˚C with the following primary antibodies from Cell Signaling Technology (Danvers, USA): Notch1 (#3608), Jagged1 (#70109), Hes1 (#11988) and β-actin (#3700), and all were used at a 1:1000 dilution. After washing three times, the membranes were incubated with goat anti-mouse (#7076) or anti-rabbit (#7077) HRP-conjugated secondary antibodies (Cell Signaling Technology, USA). The signals were analyzed using an ECL detection kit (Cat. 32106, Pierce Biotechnology, USA).

**Table 1:** Primer sequences for quantitative real-time PCR


Gene	Forward sequence	Reverse sequence

*MiR-200b-3p*	5´-GCGGCTAATACTGCCTGGTAA-3´	5´-GTGCAGGGTCCGAGGT-3´
*Notch1*	5´-GCACGTGTATTGACGACGTTG-3´	5´-GCAGACACAGGAGAAGCTCTC-3´
*GAPDH*	5´-CCAGGTGGTCTCCTCTGA-3´	5´-GCTGTAGCCAAATCGTTGT-3´
*U6*	5´-CTCGCTTCGGCAGCACA-3´	5´-AACGCTTCACGAATTTGCGT-3´


### Enzyme-linked immunosorbent assay

After the indicated treatments, the supernatants from the HUVECs were centrifuged at 1,000 x g for 5 minutes at 4˚C prior to enzyme-linked immunosorbent assay (ELISA). The levels of VEGF (#DVE00) were measured using commercial ELISA kits (R&D Systems, Inc., Minneapolis, USA) according to the manufacturer’s protocol. Each sample was evaluated in triplicate.

### Cell viability assay

A Cell Counting Kit-8 (CCK8, Cat. CK04, Dojindo, Japan) assay was used to detect cell viability. Briefly, after the indicated treatment, 1×10^4^ cells were seeded into 96- well plates, and CCK-8 solution was added to each well. After 2 hours of culture, the absorbance was measured at 450 nm using a spectrophotometer. 

### Wound-healing assay

The protocol was carried out as previously described (26). Briefly, HUVECs were seeded with the indicated treatments and then transfected with the indicated miRNA negative control or miRNA. Forty-eight hours later, the attached cells were scratched with a 10 μl pipette tip, and images were captured under a microscope at 0 hours after the scratch. The plates were returned to the incubator and cultured for 24 hours. Then, another set of images of the same wounds was captured. The wound area was measured with ImageJ and was normalized and presented as a percentage of the initial wound measured at 0 hours.

### Transwell assay

A Transwell assay was performed according to a reported protocol (27). After the indicated treatment, a total of 5×10^5^ HUVECs were suspended in a serum-free culture medium and seeded into the upper insert of a 12- well Transwell plate (Cat. 3401, Corning Incorporated, USA), with or without Matrigel pretreatment. Medium with serum was added to the lower chamber. The plate was incubated in the incubator for 8 hours. Cells remaining in the upper insert were removed using cotton swabs, and the migratory cells were fixed with 4% paraformaldehyde for 10 minutes. After washing with PBS 3 times, the cells were stained with a crystal violet solution. Images were captured using brightfield microscopy (Olympus, Tokyo, Japan) and quantified.

### *In vitro* Matrigel angiogenesis assays

*In vitro* Matrigel angiogenesis assays were performed to test the angiogenic abilities of cells. Briefly, 24 hours after the indicated treatment, HUVECs were seeded on normal Matrigel (Cat. 356234, BD Biosciences, USA) in 96-well plates (Sigma, USA). Tube lengths and branches were measured and quantified by ImageJ software. 

### Dual-luciferase reporter assay

The 3’UTR region of Notch1 mRNA was amplified by PCR and cloned into a pGL3 vector (Promega, USA). HEK 293T cells were seeded into 24-well plates and were then cotransfected with a vector carrying either the wild type or mutant Notch1 3′-UTR and either a miR200b mimic or a miR-negative control. Transfections were performed using Lipofectamine 2000 according to the manufacturer’s protocol. Finally, luciferase activities were measured using a dual-luciferase reporter gene assay kit (Cat. E1910, Promega, USA).

### Statistical analysis

Statistical analysis was performed using GraphPad Prism 5. All experiments were conducted at least three times. All data are presented as the mean ± SD. The data were analyzed by one-way ANOVA, followed by Tukey’s post hoc test or an independent sample t test. P<0.05 was considered statistically significant.

## Results

### miR-200b was upregulated in high glucose-treated HUVECs

**Fig.1:**
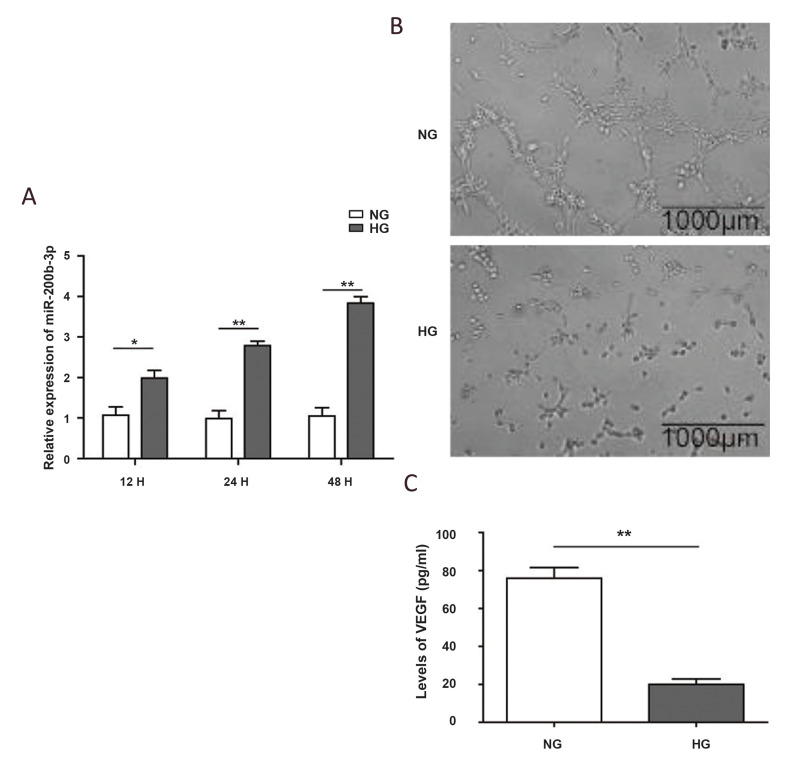
The high-glucose treatment induced the expression of miR-200b and impaired angiogenesis. **A.** Quantification of miR-200b by realtime PCR in Human umbilical vein endothelial cell (HUVECs) grown in normal glucose (NG) or high-glucose (HG) conditions for 12, 24, and 48 hours. U6 was used as an internal control for normalization. **B. **Representative images of HUVECs under different conditions during the *in vitro* angiogenesis assay. **C.** Quantification of secreted Vascular Endothelial Growth Factor (VEGF) from HUVECs, as determined by enzyme-linked immunosorbent assay (ELISA) after the indicated treatment. (N = 3). *; P<0.05, **; P<0.01, and H; Hour.

To investigate the role of miR-200b in endothelial cell dysfunction, we first tested its expression in HUVECs grown in high glucose conditions. As shown in Figure. 1A, the miR-200b level was significantly induced by high glucose treatment after just 12 hours (P<0.05), and it continued to increase to a level that was 2-fold greater than the initial levels after 48 hours (P<0.01).

Meanwhile, the tube formation ability of HUVECs was also impaired by high glucose treatment, as indicated by the decreased formation of tubes (Fig .1B). Unsurprisingly, other genes related to angiogenesis also changed, which is exemplified by the decrease in VEGF (Fig .1C, P<0.01). In addition, high glucose treatment dramatically increased the level of IL-1β (Fig .S1, See supplementary online information at www.celljournal.org). These results suggest a potential role for miR-200b in preventing endothelial cell angiogenesis *in vitro*.

### Knockdown of miR-200b promoted the proliferation and angiogenesis of HUVECs grown in high glucose conditions

**Fig.2:**
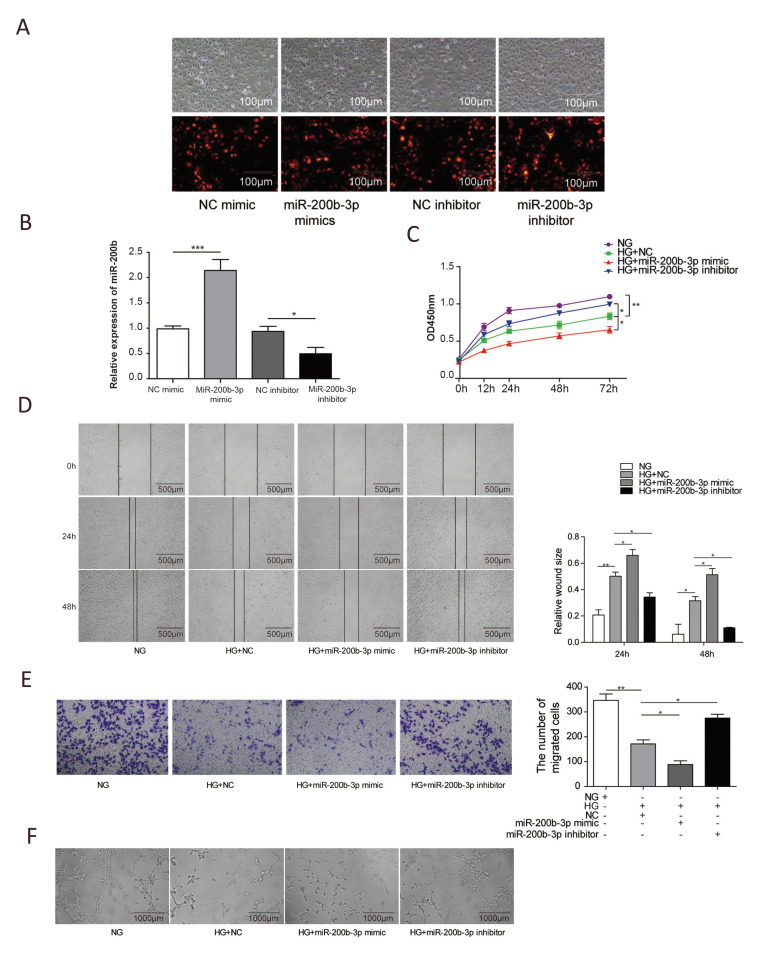
miR-200b affected the angiogenesis ability of Human umbilical vein endothelial cell (HUVECs). The HUVECs cells were transfected with NC mimic, miR200b mimics, NC inhibitor, miR-200b inhibitor, and miR-200b transfection efficiency were analyzed by **A.** IF imaging and **B.** Real-time PCR. **C.** Quantification of HUVEC viability after the indicated treatment, as determined by CCK-8 assays. **D. **Representative images of HUVECs after the indicated treatments during the wound healing assay. **E. **Typical images and quantification of HUVECs with different treatments during the migration assay. **F. **Representative images of HUVECs under different conditions during the *in vitro* angiogenesis assay. (N = 3). NG; Normal glucose, HG; high-glucose, *; P<0.05, and **; P<0.01.

To further explore the function of miR-200b in wound healing, we knocked down or overexpressed miR-200b and subsequently studied how it affected the cellular activity of endothelial cells. miR-200b knockdown decreased miR-200b expression levels, while its overexpression increased miR-200b expression levels, indicating miR200b knockdown and overexpression were transfected successfully (Fig. 2A and B). As shown in Figure.2C, high glucose treatment significantly inhibited the proliferation of HUVECs compared to cells grown in normal glucose conditions (P<0.01).

Moreover, when the miR-200b mimic was added, cell proliferation was further decreased (P<0.05). However, treatment with a miR-200b inhibitor remarkably rescued the impaired proliferation ability that was induced by high glucose treatment (P<0.05). In addition to cell proliferation, the migration ability of HUVECs was also affected. As demonstrated by wound healing and Transwell assays shown in Figure. 2D and E, compared to normal glucose conditions, high glucose treatment obviously inhibited the migration capacity of HUVECs (P<0.01) and further suppressed migration when combined with miR200b overexpression (P<0.05). When miR-200b was suppressed by its inhibitor, the ability of the cells to migrate recovered significantly (P<0.05). The tube formation ability of HUVECs was also investigated. High glucose treatment alone or in combination with miR-200b overexpression dramatically impaired the tube formation ability, which was recovered by treatment with the miR-200b inhibitor (Fig .2F). These data indicate that miR-200b can affect the proliferation, migration, and tube formation ability of endothelial cells.

### Notch1 was a direct target of miR-200b

**Fig.3:**
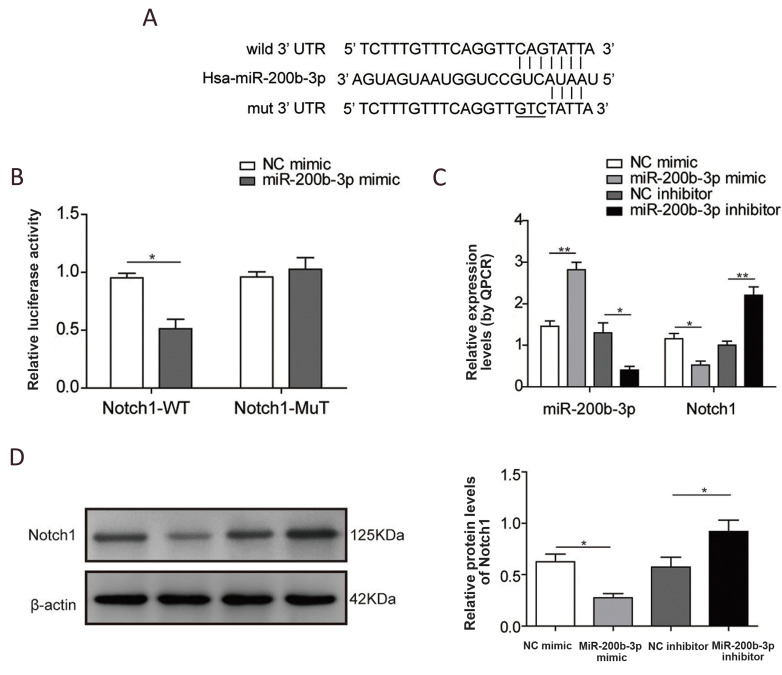
miR-200b directly targeted Notch1. **A. **Predicted miR-200b binding sites and induced mutations in the Notch1 3’UTR. **B.** Luciferase reporter assays show miR-200b-targeted Notch1 mRNA. **C.** miR-200b and Notch1 mRNA levels in Human umbilical vein endothelial cell (HUVECs) after the indicated treatments were quantified by real-time PCR. **D. **Representative images and quantification of Notch1 protein expression in HUVECs. (N=3), *; P<0.05, and **; P<0.01.

Intriguingly, as one of the most important mediators in the wound healing process, the Notch pathway is potentially regulated by miR-200b. As shown in Figure.3A, we first performed prediction searches using StarBase to identify targets of miR-200b, which indicated that miR-200b may directly target the 3’UTR region of Notch1. To further determine whether miR200b could target Notch1, we conducted a luciferase reporter assay, where the reporter contained either a wild type or a binding site mutated Notch1 3’UTR. As shown in Figure.3B, miR-200b remarkably inhibited luciferase activity from the Notch1 wild type 3’UTR (P<0.05) vector, but it did not have the same effect on the mutant. Consistently, when miR-200b was overexpressed in HUVECs, both the mRNA (Fig .3C) and protein (Fig .3D) of Notch1 were significantly suppressed (P<0.05). The downregulation of miR200b by treatment with its inhibitor increased Notch1 expression (Fig.3C and D, P<0.05). Taken together, these results demonstrate that miR-200b could target and inhibit Notch1 directly in endothelial cells.

### Inhibition of miR-200b could activate the Notch pathway and angiogenesis.

**Fig.4:**
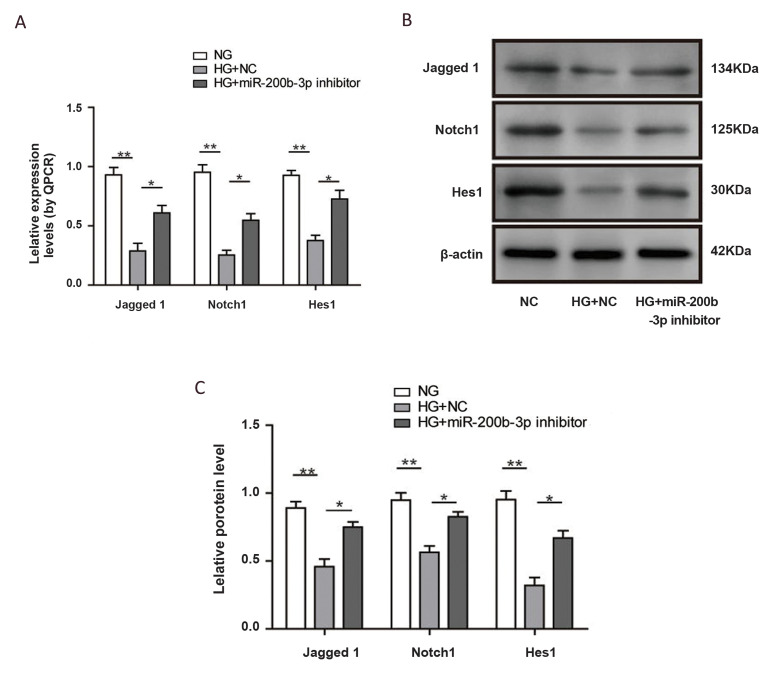
Inhibition of miR-200b could activate the Notch pathway. **A.** Quantification of Human umbilical vein endothelial cell (HUVEC) mRNA levels by realtime PCR. **B.** Representative images and quantification of Notch pathway protein expression in HUVECs. (N=3). NG; Normal glucose, HG; High-glucose, *; P<0.05, and **; P<0.01.

**Fig.5:**
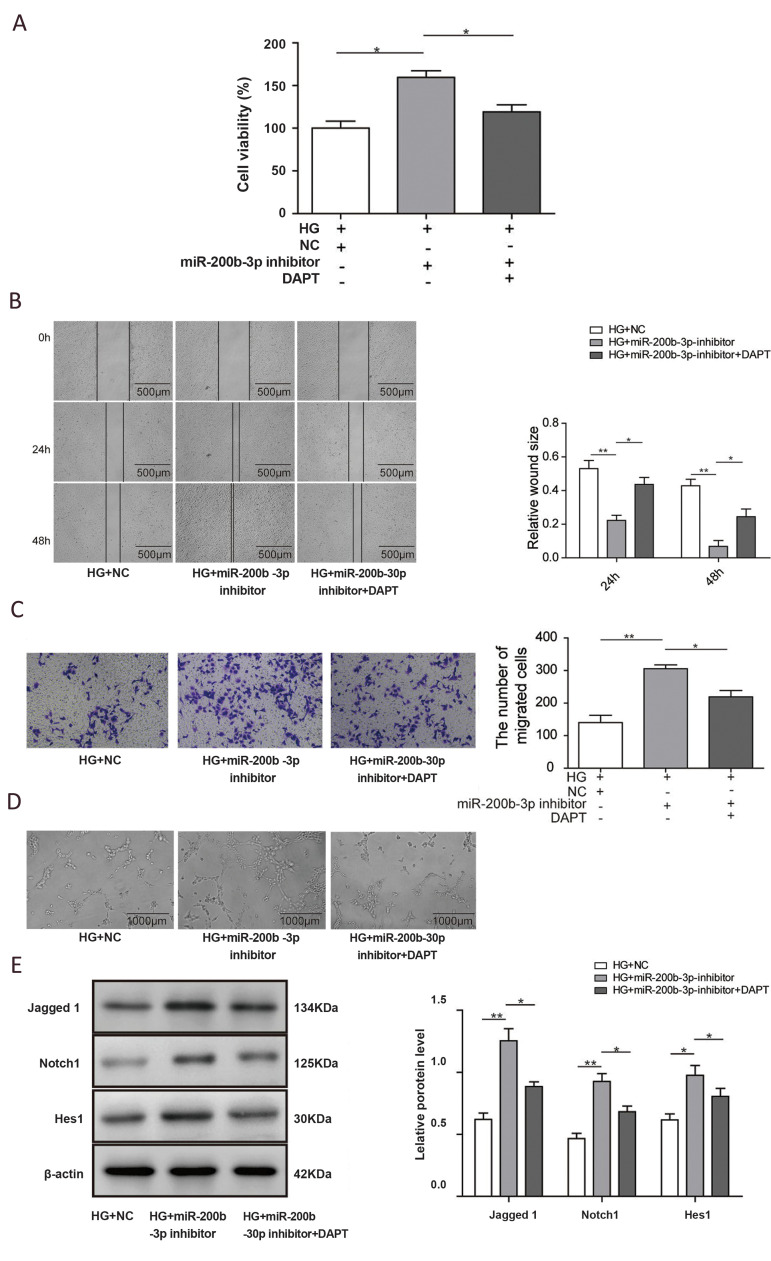
miR-200b affected angiogenesis by regulating Notch1. **A.** Quantification of human umbilical vein endothelial cell (HUVEC) viability was measured by CCK-8 assay after the indicated treatments. **B. **Representative images of HUVECs after the indicated treatments during the wound healing assay. **C. **Typical images and quantification of HUVECs with different treatments during the migration assay. **D.** Representative images of HUVECs under different conditions during *in vitro* angiogenesis assays. **E.** Representative images and quantification of Notch pathway protein expression in HUVECs. (N=3). NG; Normal glucose, HG; High-glucose, *; P<0.05, and **; P<0.01.

To evaluate whether miR-200b could regulate the Notch signaling pathway, we next examined downstream targets of the Notch pathway. Consistent with Notch1 expression, the mRNA and protein levels of Jagged1 and Hes1 were dramatically decreased by high glucose treatment (P<0.01), and they were recovered by miR200b inhibition (P<0.05, Fig.4A and B). To further determine the role of the Notch pathway in miR-200bmediated wound healing, we combined miR-200b downregulation with the Notch pathway inhibitor N- [N-(3,5-difluorophenacetyl)-l-alanyl]-S-phenylglycine t-butyl ester (DAPT) in high glucose conditions. Unsurprisingly, Notch pathway inhibition significantly repressed proliferation, which was upregulated by miR200b suppression (Fig .5A, P<0.05). Consistently, DAPT treatment could also obviously rescue the cell migration that was induced by miR-200b inhibition, as demonstrated by wound healing and Transwell assays (Fig.5B and C, P<0.05). For the tube formation function, treatment with the miR-200b inhibitor significantly increased the number of tubes formed by HUVECs, which was then decreased when DAPT was added. Downstream targets of the Notch pathway were also analyzed. As shown in Figure.5E, the protein levels of Jagged1, Notch1, and Hes1 were significantly upregulated in HUVECs grown in highglucose conditions following the treatment with the miR-200b inhibitor. When DAPT treatment was added, their expression decreased (P<0.05). These data strongly indicate that inhibition of miR-200b could stimulate angiogenesis of endothelial cells by activating the Notch pathway

## Discussion

Impaired wound healing is a major complication in diabetes patients, leading to morbidity and death (28). Skin wounds in diabetics have been linked to impaired antimicrobial activity in leukocytes, altered blood flow, and abnormal inflammatory state, all of which are related to the dysfunction of endothelial cells (29, 30). Recently, various methods have been used to treat diabetic wounds, such as tissue regeneration by stem cells or progenitor cells and administration of growth factors (31). However, the results have been very limited; since wound healing is a complex process, it is difficult to treat by targeting a single process. Due to their function in regulating multiple targets and pathological conditions, miRNAs have been considered promising therapeutic targets. The role of miRNAs in tumour angiogenesis has been widely explored, raising the potential for their use in wound healing.

Several studies have demonstrated the involvement of miRNA dysregulation in the angiogenesis process of diabetes mellitus. In a diabetic rat model, miR-320 suppressed the angiogenic response of microvascular endothelial cells by targeting insulin-like growth factor 1 (IGF1)(32). miR-93 was reported to be downregulated by high glucose treatment, and it also was found to suppress angiogenesis by targeting VEGF(33). Inhibition of miR503 could stimulate angiogenesis in diabetic ischaemic muscle by upregulating cyclin E1 (34). miR-200b has been found to inhibit angiogenesis in tumour development by targeting interleukin-8 and CXCL1, which are secreted by cancer cells (40).

Moreover, miR-200b has also been reported to play an important role in endothelial cell function. Loss of miR-200b could enhance cell motility by activating epithelial-mesenchymal transition (EMT) (22). Additionally, transient inhibition of miR200b in endothelial cells was sufficient to enhance angiogenesis during skin wound healing process (25). To explore the role of miR-200b in the wound healing process, we established a high glucose treatment assay and found a resultant upregulation of miR-200b in endothelial cells, which in turn negatively impacted proliferation, migration, and tube formation in these cells. Most importantly, the downregulation of miR200b by treatment of cells with an inhibitor could significantly rescue the high glucose treatment-induced suppression of division, mobility, and angiogenesis in endothelial cells. While high-glucose treatment has been demonstrated to diminish the angiogenesis ability of endothelial cells by altering their biochemical and biophysical properties (36), our findings reveal the underlying molecular mechanism; further, our data are consistent with the reported function of miR-200b in other disease models.

It is well established that the Notch pathway is critical for wound healing. Overexpression of Jagged1, a Notch ligand, in endothelial cells accelerated the wound healing process (37). Moreover, blocking the Notch pathway impaired wound healing by affecting the inflammatory response through the regulation of macrophages (20). Previous studies have demonstrated that miR-200b could regulate the Notch pathway in tumours by targeting Notch1 and suppressing tumour metastasis (38). The Notch ligands Jagged1 and Jagged2 were also found to be regulated by miR-200b in metastatic prostate cancer cells (39) and lung cancer (40). In the current work, Notch1 was found to be directly targeted for posttranscriptional regulation by miR-200b in endothelial cells. With the increase in miR-200b expression induced by high glucose treatment, Notch1 levels, and Notch pathway activity were significantly repressed.

On the other hand, miR-200b inhibition was proven to activate the Notch pathway and the wound healing process, which was blocked by treatment with DAPT, a Notch pathway inhibitor. Intriguingly, treatment with the miR-200b inhibitor could only partially return the expression of Notch pathway-related genes to the levels of expression observed control group, indicating the possibility that other angiogenesis-associated targets are regulated by miR-200b in high glucose conditions. This emphasizes the role of miR-200b in regulating the Notch pathway during diabetic wound healing.

## Conclusion

In summary, this work demonstrates that miR200b is upregulated by high glucose treatment in endothelial cells, impairing the wound healing process by suppressing cell proliferation, migration, and angiogenesis. The knockdown of miR-200b is sufficient to restore HUVEC dysfunction by stimulating the Notch pathway, which is shown to be directly regulated by miR-200b and plays a critical role in the wound healing process. Our findings illustrate the function of miR-200b in wound healing and highlight the potential of miR-200b as a promising therapeutic target in the treatment of diabetic complications. However, the current work is still based on an *in vitro* assay mimicking diabetic conditions by high glucose treatment. Further study on animal models is required to explore the function of miR-200b in the diabetic wound healing process and determine its potential as a treatment target.

## Supplementary PDF


