# Supplementary material for: Inhibition of miR-200b Promotes Angiogenesis in Endothelial Cells by Activating The Notch Pathway
Source: Cell J. 2021 Mar 1;23(1):51–60. doi: 10.22074/cellj.2021.7080 (PMC7944128; doi:10.22074/cellj.2021.7080)
Supplement: Supplementary file 1 [file Cell-J-23-51-s01.pdf]

CELL JOURNAL (Yakhteh)

Supplementary Information for

Inhibition of miR-200b promotes angiogenesis in endothelial cells by activating the Notch pathway

Tie-Ying Qiu, M.M.[1], Jin Huang, M.D.[1], Li-Ping Wang, M.M.[1], Bi-Song Zhu, M.D.[2*]

1. Clinical Nursing Teaching and Research Section of the Second Xiangya Hospital, Changsha 410011, P.R. China
2. Organ Transplant Center, Xiangya Hospital, Central South University, Changsha 410008, P.R. China

**Corresponding Address: Organ Transplant Center, Xiangya Hospital, Central South University, Changsha 410008, P.R. China*
Email: 277364307@qq.com

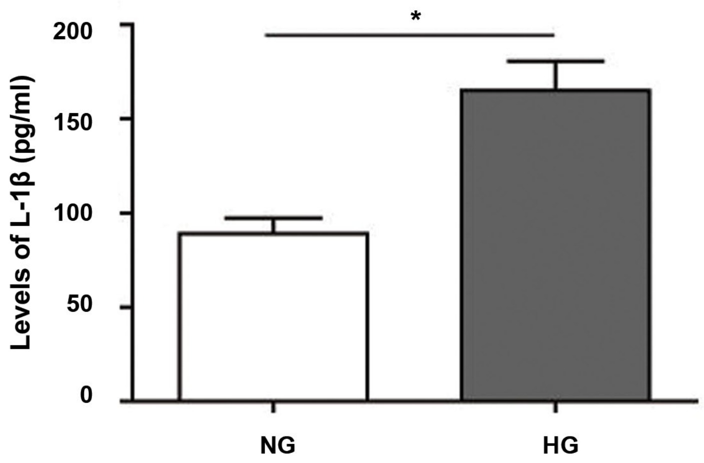

Fig.S1: Quantification of secreted Interleukin-1 beta (IL-1β) from HUVECs, as determined by enzyme-linked immunosorbent assay (ELISA) after the indicated treatment.
